# Parameters of anesthesia/sedation in children receiving radiotherapy

**DOI:** 10.1186/s13014-015-0363-2

**Published:** 2015-03-11

**Authors:** Kevin P McMullen, Tara Hanson, Jennifer Bratton, Peter A S Johnstone

**Affiliations:** IU Health Proton Therapy Center, Bloomington, Indiana; Department of Radiation Oncology, Indiana University School of Medicine, Indianapolis, Indiana USA; Department of Radiation Oncology, Moffitt Cancer Center & Research Institute, Tampa, Florida USA

**Keywords:** Pediatric, Radiotherapy, Anesthesia requirement, Gender, Age

## Abstract

**Background:**

Previous reports establish low risk of complications in pediatric treatments under anesthesia/sedation (A/S) in the outpatient setting. Here, we present our institutional experience with A/S by age and gender in children receiving daily proton RT.

**Methods:**

After Institutional Review Board approval, we reviewed our center’s records between 9/9/2004 and 6/30/2013 with respect to age and gender of A/S requirement in our pediatric patients (defined as patients ≤18 years of age).

**Results:**

Of 390 patients treated in this era, 182 were girls. Children aged ≤3 invariably required A/S; and by age 7–8, approximately half of patients do not. For pediatric patients ≥ 12 years of age, approximately 10% may require A/S for different reasons. There was no difference by gender.

**Conclusions:**

Beyond age 3, the requirement for A/S decreases in an age-dependent fashion, with a small cadre of older children having difficulty enough with sustained immobilization that A/S is necessary. In our experience, there is no difference in A/S requirement by gender.

## Introduction

The outpatient delivery of radiation therapy (RT) for children receiving daily anesthesia and/or sedation presents significant challenges. We have previously reported that repetitive daily anesthesia/sedation (A/S) may be provided safely to these children treated at free standing centers remote from hospital support, with low risk of complication or hospitalization [1]. We have also reported on the typical venous access devices and complication rates of each associated with their use in this milieu [2].

Factors requiring use of A/S to facilitate RT are poorly defined, varying on an individual basis. Such factors include, but may not be limited to: airway management, postoperative complications, cognitive function, emotional maturity, pain, musculoskeletal deformities, claustrophobia, and fear of the environment of treatment. Patients requiring RT frequently are immobilized in restraint devices that create anxiety that may require A/S to ensure accurate RT delivery.

Indiana University Health Proton Therapy Center (IUHPTC) has established expertise in treating pediatric patients in the outpatient setting [1]. In center, pediatric patients are assessed for A/S by the attending radiation oncologist after evaluation and consultation with the patient and caregiver(s). The decision to pursue either general anesthesia or intravenous sedation in the delivery of care adds several levels of complexity to the care course including anesthesia risks, risks of daily manipulation of venous access devices [3-6], increased treatment delivery time, requirement for anesthesia recovery teams and space, increased cost due to anesthesia billings, and scheduling constraints on the entire department due to patient NPO needs requiring early treatments.

Treatment centers offering A/S have little formal guidance on selection of patients short of physician experience based on the treatment and setup required. However, staff planning, scheduling of other patients and center anesthesia services all require significant advance notice of A/S requirements. To contribute to the scarce literature on the subject, this manuscript describes practical aspects of our experience with RT to pediatric patients. We have used these data to adapt A/S use and optimize pediatric scheduling at our facility.

## Methods

After approval by the Indiana University Institutional Review Board, the records of the IUHPTC were screened for pediatric (≤18 years of age) patients receiving A/S with proton therapy at our center between 9 September 2004 and 30 June 2013. Subjects who underwent daily A/S during any portion of their RT were identified and classified by gender and age. Particulars of the children receiving GA have been published previously [1].

## Results

**Figure 1:**
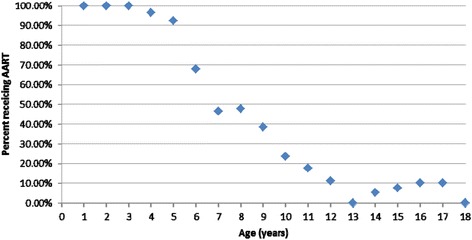
**Graphical representation of percentage of patients requiring GA for radiotherapy treatment (Y-axis) based on age at time of treatment (x-axis).**

390 pediatric patients were treated within the study period, 182 (47%) were girls. Patient demographics are collated in Table 1 and Figure 1.

**Table 1:** **Patient demographics by age and gender at time of radiotherapy delivery**

**Age**	**# Boys**	**Boys w/A/S**	**# Girls**	**Girls w/A/S**
≤**1**	9	9	12	12
**2**	18	18	25	25
**3**	15	15	17	17
**4**	13	13	16	15
**5**	18	17	8	7
**6**	15	6	10	8
**7**	16	8	12	5
**8**	9	4	14	7
**9**	5	2	8	3
**10**	8	3	9	1
**11**	8	1	9	2
**12**	11	0	7	2
**13**	15	0	5	0
**14**	9	0	10	1
**15**	15	2	12	0
**16**	13	1	7	1
**17**	11	1	9	1
**18**	0	-	1	0

Parameters of the study population.

Data review revealed 100% of patients aged ≤3 years of age required A/S. This decreased by age, with about half of patients aged 7–8 being able to undergo RT without support. Pediatric patients aged ≥13 years still had ~10% rate of A/S requirement, and that there was no statistically different rate of A/S use by gender.

## Discussion

Due to complexity of care, pediatric RT treatments are typically delivered by facilities associated with academic teaching hospitals and tertiary care children’s hospitals. As discussed in our previous reviews of our pediatric practice [1,2], the location of IUHPTC over 50 miles away from Riley Children’s Hospital and 3 miles from IU Health Bloomington Hospital mandates that structured teamwork and training be continuously practiced. The addition of A/S to an already-challenging pediatric RT treatment course adds marked complexity. The burden on patients of NPO requirements, and the potential disruption to clinic treatment flow and efficiency mandates extensive pre-planning, and proton therapy centers must individually establish efficient workflow to decrease the burden on the entire system [7,8]. Thus, we performed this analysis to identify age and gender patterns as a first step in establishing patterns of A/S use by demographic factors. While we did not specifically track adverse events in this population dating to 2004, informal discussion with staff personnel did not reveal additional events besides those previously reported [1].

These data reveal that GA use is necessary in all patients age 3 and younger, with an age-dependent decrease until age 13 (Figure 1). These data are consistent with limited data in the anesthesia literature assessing pre-operative anxiety. In a prospective study by Kim and associates [9], age, type of parent or guardian, number of siblings, waiting time and score on the modified Yale Preoperative Anxiety Scale (mYPAS) were assessed for predicting need for pre-operative sedation for anxiety in children prior to general anesthesia (GA). In multivariate analysis, age, mYPAS score and waiting time were significant factors related to anxiety levels requiring preoperative sedation. The authors noted the same age-dependent pattern of decreasing need for pre-operative sedation and also the same lack of difference by gender as noted in our series. Zeev and colleagues [10] identified parent anxiety, child temperament, age and previous medical experiences as factors increasing child anxiety before GA. Methods to decrease child anxiety should be considered to facilitate their RT without A/S. Such strategies may be employed with little burden to the system.

Non-medication strategies to relieve preoperative anxiety and decrease the need for A/S have been reported. These strategies vary widely and include cartoon viewing [11], hand-held video game play [12], and presence of clown doctors [13]. While novel, these are admittedly impossible to correlate with daily RT delivery. Other strategies such as Child Life staffing [14] to employ interactive strategies have been reported as successful in the diagnostic imaging milieu and merit prospective evaluation in pediatric RT delivery.

One simple parameter that should be considered when considering A/S in any pediatric RT patient is the complexity of the setup. Our clinic has extensive experience with supine craniospinal (CSI) RT. In our prior time study of the development of the technique [15], we documented that, even after documentation of team facility with any patient, average setup and treatment times for the four required fields remained just under one hour. If patients require CSI, that benchmark should be kept in mind. Although most older children will be able to tolerate one hour without A/S, about half of children 10 or older requiring A/S in our experience did so for CSI fields. However, the converse should be kept in mind: younger patients requiring single fields or less complex setups may be able to lie still without A/S for the shorter times involved. This allows the clinic to reserve the limited anesthesia resources for the children that really need them. In our experience, treating pediatric patients in general is done at a considerable financial loss [16]. Minimizing the costs inherent in A/S can only ameliorate that problem.

## Conclusions

In pediatric RT, A/S adds substantial burden to the patient and system, mandating factors predicting its necessity be identified. In children receiving daily RT, need for A/S decreases in an age-dependent fashion after the age of 3, without a gender effect. About 10% of children 12 or older still require A/S.
